# Teasing in Outpatient Clinical Interaction in China: Managing Epistemic and Deontic Authorities

**DOI:** 10.1111/hex.14114

**Published:** 2024-06-21

**Authors:** Ying Gao, Shuai Zhang, Wen Ma

**Affiliations:** ^1^ Center for Clinical Neurolinguistics, School of Foreign Languages and Literature Shandong University Jinan China; ^2^ College of Foreign Languages Shandong Agricultural University Taian China; ^3^ School of Foreign Languages Yantai University Yantai China

**Keywords:** clinician–patient interaction, conversation analysis, deontic authority, epistemic authority, teasing

## Abstract

**Introduction:**

In response to evolving patient‐centred care initiatives, this study investigates teasing in Chinese outpatient interactions. By exploring how teasing impacts knowledge exchange and decision‐making processes, the research underscores its utility in enhancing patient engagement and communication efficacy, aligning with China's healthcare reform objectives.

**Methods:**

Utilizing conversation analysis, the study meticulously examines a series of outpatient interactions, identifying and analyzing instances of teasing. Through a detailed investigation of the sequential organization, design features, and interactional tasks accomplished by teasing, the research unravels the complex mechanisms through which teasing operates within clinical settings.

**Results:**

Findings reveal that teasing is a sequential accomplishment that is intertwined with institutional contingencies and embedded in overarching sequential projects of clinics. Teases often arise from subtle cues that may lead to delicate moments and are crafted with a blend of playfulness and seriousness, serving to bridge the gap between patient experiences and medical expertise. This study highlights how teasing facilitates the exchange of knowledge and influences collaborative decision‐making, marking a departure from its more casual use in everyday interactions. Teasing acts as a catalyst for a more profound comprehension of medical facts, encouraging patients to actively participate in their care by expressing concerns and preferences, thus aligning medical decisions with patient autonomy. The responses to teases vary, reflecting the complexity of clinical communication and the need for a delicate balance between humour and professionalism.

**Conclusions:**

The study underscores the significance of teasing as a multifaceted communicative practice within outpatient clinical interactions. It highlights how teasing can contribute to a more nuanced and patient‐centred approach to healthcare, enhancing the collaborative exploration of medical advice and fostering a more engaging and empathetic clinical environment.

**Patient or Public Contribution:**

This study utilized real outpatient interactions, with participants' informed consent. The analysis focused on authentic teasing exchanges between clinicians and patients, ensuring the findings reflect genuine communication practices in healthcare settings. Participants, including both healthcare professionals and patients, were informed about the study's goals and their essential role in providing insights into clinician–patient communication, contributing to a deeper understanding of interaction dynamics in outpatient clinics.

## 1 Introduction

The quality of doctor–patient interactions is paramount in healthcare, influencing patient satisfaction, adherence to medical advice, and overall health outcomes [1, 2]. These interactions skillfully balance relational and instrumental goals, fostering rapport while focusing on diagnosis and treatment [3, 4]. Within this complex interplay, commutative practices such as humour, teasing and small talk emerge as unique and understudied, functioning beyond mere social lubricants to influence rapport and decision‐making processes.

Humour in medical settings often alleviates tension and bridges the serious tasks of healthcare provision. Its use ranges from creating lighter interactions to facilitating complex medical dialogues [5, 6, 7, 8, 9]. Small talk, although seemingly casual, plays a strategic role in medical settings by easing into conversations, creating rapport, and providing gateways to more serious discussions, often setting a nonthreatening stage for the delivery of sensitive medical information [10, 11, 12]. While typically perceived as light and nonserious, small talk can serve critical communicative functions that indirectly contribute to clinical outcomes, thus blending casual interaction with serious undertones when necessary. Unlike humour and small talk, teasing, while sharing a seemingly casual tone, engages more directly with medical complexities and addresses patient concerns more pointedly, offering a unique lens through which to view relational and institutional dynamics whining clinical interactions.

This study delves into the intricate role of teasing in the clinician–patient interactions within Chinese outpatient settings, a context where the traditional hierarchical nature of relationships and the brevity of interactions present unique communicative challenges. The shift towards patient‐centred care in these settings underscores the need for nuanced communication practices [13, 14, 15, 16].

Teasing in interpersonal communication is often a blend of jest and provocation, a nuanced interplay that goes beyond mere humour to render certain actions conditionally relevant. It facilitates both affiliation and subtle behavioural influences, based on a delicate balance of rapport and provocation [17, 18, 19]. In clinician–patient interactions, teasing, whether initiated by clinicians, patients, or companions, adapts to the distinct roles and relationships, maintaining a light‐hearted and nonaggressive nature, carefully modulated to respect professional and therapeutic boundaries, while still achieving a range of interactional tasks.

The cultural context of China, with its rapid evolution towards more collaborative patient–clinician dynamics, makes the study of teasing particularly relevant for improving healthcare interactions. This research aims to elucidate how teasing, shaped by and shaping the roles of those involved, contributes to the delicate dance of clinician–patient communication in China, highlighting its potential to enhance patient engagement and satisfaction in a patient‐centred care paradigm [13, 15].

### 1.1 Teasing: Sequence, Design, and Interactional Roles

Teasing is a multifaceted social action that blends humour with seriousness, serving diverse functions across both personal and institutional contexts [17, 19, 20]. In conversation analysis (CA), teasing is examined for its detailed linguistic and nonverbal cues, which demonstrate its adaptability and function across different settings, highlighting its role in navigating complex social dynamics [18, 21, 22]. This study focuses on three salient aspects of teasing within the CA framework: sequence, design, and action, particularly how these elements play out in clinician–patient dialogues.

The ‘sequential structure’ of teasing in CA involves an orderly arrangement of conversational turns that start with a ‘teasable’—a mild deviation from norms that invites a ‘tease’, a playful challenge emphasizing contingency and context‐sensitivity [18, 19, 23, 24]. Receipts of a tease can vary widely, from laughter and acknowledgement of the jest to serious engagement or even resistance, reflecting the nuanced capacity of teasing to serve both (dis)affiliative functions within interactions [17, 19]. In healthcare, where professional and therapeutic stakes are high, teasing must be carefully modulated to align with the institutional objectives of effective communication and patient care [5, 7, 25, 26].

Design features of teasing, such as hyperbole, irony, and rhetorical questions, along with nonverbal cues like tone, pitch, and laughter, signal its nonserious intent and help differentiate teases from more serious communicative acts. These elements are crucial for ensuring that teasing serves its intended purpose within professional settings without breaching decorum or patient comfort [19, 27, 28, 29].

It's important to note that in institutional interactions, such as clinician–patient dialogues, ‘action’ encompasses not only the act of teasing itself but also the various interactional roles it may assume, furthering specific institutional tasks. Teasing enhances relational dynamics and facilitates communication, capable of strengthening social bonds or managing professional decorum in healthcare. It balances humour and provocation, fostering engagement and empathy within medical consultations [5, 25]. The dual nature of teasing as both an affiliative and corrective mechanism underscores its value in reinforcing and challenging conversational norms, helping balance the delivery of medical information with empathetic patient engagement [7, 9].

### 1.2 Epistemic and Deontic Authority in Clinical Interactions

The negotiation of knowledge and authority is pivotal in clinician–patient interactions, involving the exchange of medical advice and decision‐making about treatment options. Drawing on the work of Heritage [30, 31, 32] and Stevanovic and Peräkylä [33], this study explores the dynamics of epistemic and deontic authority. Epistemic authority concerns the rights to know and convey knowledge within a domain, primarily held by clinicians due to their medical expertise, while patients contribute their personal experiences and insights, thus forming their own epistemic territories [34].

Clinicians guide treatment recommendations with epistemic primacy, while patients often bring valuable experiential knowledge, sometimes challenging the clinicians' advice [35]. This interplay between professional expertise and personal experience underscores the collaborative nature of health decision‐making. Deontic authority involves the right to make or influence decisions and actions [33]. While clinicians propose treatments leveraging their epistemic authority, patients possess the ultimate deontic right to accept or reject these recommendations [36].

In contexts like China, where healthcare settings are evolving towards more patient‐centred care, understanding the interplay between these forms of authority is crucial. This dynamic is essential for both parties to navigate medical consultations effectively, making informed decisions that respect both medical expertise and patient autonomy. Jakovljevic et al. [15, 37]. Highlighting the sequential intricacies, multifaceted nature and the diverse roles of teasing, this research not only enriches our insight into medical communication but also underscores the value of adaptive conversational practices in handling sensitive interactions effectively amidst ongoing healthcare reforms and shifts towards patient‐centred care paradigms [13, 38].

## 2 Method

This study's data set originated from a research initiative at a university in Shandong Province, China. It includes 4.5 h of audio‐recorded orthopaedic outpatient consultations at a tertiary hospital, covering 77 sessions ranging from 26 s to 11 min in length. Informed consent was obtained from all participants before recording, adhering to ethical research standards, and the study was approved by the Ethics Committee of the School of Basic Medical Sciences at Shandong University. Participant anonymity and data confidentiality were ensured, with pseudonyms used to protect identities.

Due to the nature of the data, our analysis focused primarily on linguistic and paralinguistic cues, excluding visual cues which are pivotal for fully understanding the context of teasing [18, 22]. Transcriptions were conducted using Gail Jefferson's system [39, 40], adapted to accommodate specific features of spoken Chinese. The transcription format included standard Chinese orthography with annotated prosodic features, Pinyin transcription, morpheme‐by‐morpheme glosses, and English translation, ensuring an accurate capture of communicative nuances (see Supporting Information S1: Appendix for transcription conventions).

Our methodological framework is grounded in CA, as outlined by Sidnell and Stivers [41], which emphasizes analyzing the sequential unfolding of interactions from the participants' perspectives. CA focuses on interactionally relevant behaviours, avoiding assumptions about macrocontextual factors such as identity unless explicitly indicated by participants [42, 43, 44, 45, 46, 47, 48]. This approach aligns closely with the principles of CA, making it particularly valuable for examining teasing sequences and their integration within medical consultations, reflecting participants' social and cultural understandings [48]. Furthermore, the application of CA in healthcare research is well‐documented, with studies demonstrating its utility in understanding a variety of social actions (e.g., [49, 50, 51], Ma et al., 2023). These studies demonstrate CA's relevance in addressing sequential concerns such as preference organization, turn‐taking, pursuit of response, and patterns of (dis)affiliation, which are crucial to our data analysis.

In identifying teasing sequences, we adhered closely to CA's foundational analysis principles, prioritizing how participants themselves understand and navigate their interactions. Our analysis was informed by existing literature on the sequential and design characteristics of teasing, including the presence of a ‘teasable’ event, the delivery of the tease marked by specific linguistic and paralinguistic cues, and the recipient's response [18, 19, 20, 43]. These responses, ranging from laughter to more serious rebuttals, provide critical insights into how teases are perceived and the roles they play in the ongoing interaction.

Each teasing sequence identified was meticulously reviewed, with 17 sequences ultimately recognized across seven consultations. This rigorous verification process involved the research team's consensus, ensuring that our findings authentically reflected the participants' engagement and understanding [41, 42].

Our analysis focused how teasing influences clinician–patient interactions, considering both relational and instrumental tasks, and contextualized within the broader literature on teasing and doctor‐patient communication [16, 52, 53] (Shirokov, 2024). This approach illuminated the intricate role of teasing in medical interactions, highlighting how such sequences contribute to the sequential unfolding of interactions and the management of institutional goals in clinical settings [49, 51].

## 3 Analysis

Our data reveals teasing's presence across various medical phases, including history‐taking, diagnosis delivery, and treatment planning, demonstrating its flexibility and adaptability among doctors, patients, and companions [54]. Teasing's impact falls into two main categories: navigating epistemic territories and managing deontic authority, which illustrate its nuanced role in negotiating knowledge and authority within medical consultations [30, 31, 32, 33].

### 3.1 Navigating Epistemic Territories

In this category, teasing engages with the exchange and affirmation of medical knowledge, often involving medical conditions, symptoms, or lifestyle discussions. Extract 1 showcases a patient discussing the challenges associated with an external fixator after a vehicular accident. His narrative intertwines medical details with personal struggles, setting a complex scene for teasing that enhances mutual understanding. The patient expresses frustration over the delayed removal of the fixator, initially promised by a previous doctor. His response (line 1) to the doctor's assessment that it is premature to remove the fixator highlights the emotional and practical implications of the delayed medical procedure.

(1) O1957: Crying loudly

**Table:** 

01 → PAT	你把这个还得‐(.)
	nǐ bǎ zhè gè hái déi ‐(.)
	you BA this CL still have to
	*You still want (to keep) it ‐*
02 →	如果不拆的话,得带多长时间?
	rú guǒ bù chāi de huà, dé dài duō zhǎng shí jiān?
	if NEG remove CSC talk have to wear how long time
	*If it is not removed, how long will (I) wear it?*
03	(0.7)
04 → DOC	是不是当时你光‐在那里嗷嗷的在
	shì bú shì dāng shí nǐ guāng ‐ zài nà lǐ áo áo de zài
	be NEG be then you only at there ao ao CSC at
	*Were you just there going ‘ao ao’, wailing away*,
05 →	那里哭,哄你说的三‐三个月啊.
	nà lǐ kū,hǒng nǐ shuō de sān ‐ sān gè yuè a.
	there cry coax you say CSC three three CL month PRT.
	*and they coaxed you by saying ‘three months’, right?*
06	(0.4)
07 → PAT	哭倒没哭.疼[啊.
	kū dào méi kū. téng [a.
	cry actually NEG cry. pain PRT.
	*I didn't cry, through. It [pained*.
08 DOC	[你不是疼吗?
	[nǐ bú shì téng ma?
	[you NEG be pain PRT
	[*Didn't you feel painful?*
09	(0.3)
10 PAT	疼我也不哭.
	téng wǒ yě bù kū.
	pain I either NEG cry
	*Even it pained, I didn't cry*.
11	(0.3)
12 DOC	不哭,你‐你你你:如果要是:(0.2)
	bù kū, nǐ ‐ nǐ nǐ nǐ : rú guǒ yào shì :（0.2）
	NEG cry you you you you if suppose
	*You didn't cry. If ‐*
13	如果要是说就是:不是这个的话,
	rú guǒ yào shì shuō jiù shì : bú shì zhè gè de huà,
	If suppose say just be NEG be this CL ASSC talk,
	*If it was not the case*,
14	三个月那是不对的哈.（.）
	sān gè yuè nà shì bú duì de hā.（.）
	three CL month that be NEG right ASSC PRT
	*then three‐month is not correct*.
15	绝对不对的.半年都不对.
	jué duì bú duì de. bàn nián dōu bú duì.
	definitely NEG right ASSC half year all NEG right.
	*Definitely not correct. Even half a year is not correct*.
16	(0.8)
17 PAT	这十个[月了,
	zhè shí gè [yuè le,
	this ten CL month PFV.
	*It has been ten months*.
18 DOC	[你‐给你造成了
	[nǐ ‐ gěi nǐ zào chéng le
	[you‐ give you cause PFV
19	一个很大的误导啊,这是.
	yí gè hěn dà de wù dǎo a, zhè shì.
	one CL very big ASSC misleading PRT, this be.
	*This misled you a lot*.

In line 1, the patient's use of the adverb ‘dei’ (still) suggests the ongoing impact of the external fixator (‘zhege’), framing his turn as a complaint. The direct address ‘ni’ (you) implicates someone in a role of blame, highlighting the interplay of hurt and blame crucial for understanding his complaint [55]. The patient initiates a self‐repair with a cut‐off, pivoting from a complaint to a question using ‘dei’, a key moment in navigating epistemic territories [56]. This inquiry invites the doctor to contribute information, but it's interpreted as an escalated complaint rather than a straightforward question, explaining the notable silence (line 3).

The doctor responds with a blend of jest and subtlety, aligning with Drew's [19] concept of teasing, involving exaggeration and attributing category‐downgrading characteristics. Specifically, the repetition of ‘ao ao’ (mimicking loud crying) exaggerates the patient's emotional response, introduces humour that contrasts with traditional masculine norms, and highlights the atypicality of an adult man wailing away. This light‐hearted yet critical portrayal emphasizes the patient's overly dramatic behaviour. The teasing also serves as an account, subtly implying that the patient's exaggerated emotional display may have influenced the previous optimistic prognosis, thereby redistributing some responsibility back to the patient [57]. This practice engages delicately with patient expectations and professional stances, aligning with the omnirelevance of accountability [58].

The patient's response, devoid of laughter or explicit amusement, reflects observations by Emerson [59] and Mallett and A'Hern's [7] that humour may falter in contexts of resistance or high emotional distress. Despite recognizing the tease's humour, the patient's po‐faced rejection [19] signals resistance, underscoring the serious undertones and existing tension within the interaction [16]. The teasing sequence emerges from the conflict between the medical guidelines and patient's repeated requests and implied complaints, culminating in the doctor's jocular proposal met with po‐faced rejection.

The patient's continued resistance underscores his earnest plea for a resolution. The doctor's response (line 8), normalizing the alleged behaviour, evidence a shift in conversational strategy. This adjustment is further illustrated by the doctor's use of a contingency clause ‘if it not was the case’ (line 13), indicating the doctor's efforts to realign the interaction, subtly shifting the responsibility back to the prior doctor and suggesting a shared burden of the prognosis.

This interaction showcases how teasing can effectively navigate epistemic territories, redistributing responsibility while expanding the doctor's epistemic knowledge. The doctor's approach not only addresses the patient's concerns in a nonconfrontational manner but also subtly corrects misconceptions about the recovery timeline, stating ‘three‐month is not correct’ (line 14). This interaction bridges patient expectations with medical guidelines, demonstrating the doctor's empathetic communication style, which balances professional assertiveness with patient‐centred care. The insights from Extract 1 underscore how teasing, when employed judiciously, serves as a bridge between empathy and professionalism, gently challenges the patient's assertions without direct confrontation, thus easing the tension inherent in such critical discussions. This strategic use of humour respects the patient's perspective and reveals the importance of patient resistance, which can unearth deeper concerns, emphasizing the need for sensitive and adaptable communication practices.

Following Extract 1, Extract 2 resumes later in the same medical consultation, further exploring the evolving dynamics of teasing. This segment particularly highlights how teasing is employed not only by clinicians but also by patients. Such reciprocal engagement demonstrates that patients also use teasing effectively to assert their perspectives and influence the consultation's direction. This interaction underscores the flexibility and adaptiveness of teasing as a communicative tool, facilitating mutual understanding even amidst conflicts between personal experiences and professional guidelines. Through this analysis, we gain deeper insights into how both doctors and patients can use teasing to navigate the complexities of medical interactions.

(2) O1957: Knead

**Table:** 

01 → DOC	不疼吧?
	bù téng ba?
	NEG pain PRT?
	*It doesn't pain, right?*
02	(0.7)
03 PAT	肯定有点疼,但是不是很疼.
	kěn dìng yǒu diǎn téng, dàn shì bú shì hěn téng.
	definitely have a bit pain, but NEG be very painful.
	*It must hurt in some way, but it is not very painful*.
04	(1.0)
05 → PAT	叫‐你叫好腿你捏,
	jiào‐nǐ jiào hǎo tuǐ nǐ niē,
	ask you ask good leg you knead,
	*If you knead an intact leg*,
06→	你也疼啊, £对吧? £
	nǐ yě téng a,£duì ba?£
	you too hurt PRT, right PRT?
	*it will also hurt, £right?£*
07 → DOC	不一个疼法.
	bù yī gè téng fǎ.
	NEG one CL hurt way.
	*It hurts in a different way*.
08	(1.8)

In Extract 2, the interaction begins with the doctor inquiring if the patient experiences pain during a physical examination (line 1). Following a 0.7‐s gap, the patient acknowledges minor discomfort but seizes the opportunity to tease about the doctor's examination technique, suggesting through a playful hypothetical scenario that the discomfort might stem from how the examination was conducted (lines 5–6). The patient's remark maintains a light‐heartedness aimed at softening the critique, making it more palatable and maintaining a cooperative atmosphere. This teasing gently probes the examination methods without overtly challenging the doctor's competence or intentions.

The doctor's straightforward correction of the patient's hypothetical scenario (line 7), does not outright dismiss the humour but addresses the underlying concern it raises, illustrating a nuanced form of engagement rather than mere resistance [60]. This reaction suggests that while the doctor recognizes the humorous framing, he opts to steer the conversation back to professional boundaries without escalating any potential conflict. This approach underscores the doctor's commitment to maintaining a professional demeanour while also acknowledging the patient's input, balancing medical protocol with patient engagement.

In this sequence, the ‘teasable’ is the doctor's examination technique, while the ‘tease’ is the patient's playful scenario, met with a corrective response. The tease serves as a means for the patient to voice concerns in an environment where direct criticism might be less accepted. By framing his discomfort in the context of a hypothetical scenario, the patient normalizes his condition and invites the doctor to reconsider the examination technique without direct confrontation. The doctor's response, therefore, can be seen as an effort to validate the patient's experience while also reaffirming the professional standards expected in such interactions.

This nuanced exchange highlights the adaptive use of teasing as a communicative tool within medical dialogues, bridging professional expertise and personal experience. It demonstrates how both parties use humour to navigate the consultation, enhancing mutual understanding and maintaining a collaborative relationship. In subsequent interaction, as we observe in Extract 3, teasing continues to play a pivotal role, with a companion's intervention during the medical history‐taking phase adding depth and further illustrating how teasing can enrich dialogue and understanding in clinical settings.

(3) O1915: Dozens of years

**Table:** 

01 DOC	这种wa‐歪歪着, (0.3)多长时间啦?
	zhè zhǒng wa‐ wāi wāi zhe, (0.3) duō zhǎng shí jiān la?
	This kind cur‐ curved DUR (0.3) how long time PFV
	*How long is the curvature (of the spine)?*
02 → PAT	歪歪着,好几年啦.[在我
	wāi wāi zhe, hǎo jǐ nián la.[zài wǒ
	curved DUR great several year PFV at I
	*The curvature has been several years. When I (was)…*
03 DOC	[好几年啦?
	[hǎo jǐ nián la?
	[great several year PFV
	*Several years?*
04 PAT	昂.
	áng.
	*Eh*.
05	(0.6)
06 → COM	还好几年了,
	hái hǎo jǐ nián le,
	still great several year PFV,
	*It cannot be several years*.
07 →	这得好几十年.
	zhè déi hǎo jǐ shí nián.
	this must great several ten year.
	*It has to be dozens of years*.
08 → PAT	昂[:.好几十::]年£是[不假哩.] £
	áng [:. hǎo jǐ shí ::] nián £ shì [bù jiǎ lī.] £
	eh great several ten year be N fake PRT
	*Eh. Dozens of years*. £*It's not fake*. £
09 → COM	[↑Hah hehhh.] [↑Hah heheh]h.
10	(1.5)
11 PAT	非锅锅着,这个腰恁.
	fēi guō guō zhe, zhè gè yāo nèn.
	must arch DUR this CL waist PRT.
	*The back must be stooped itself*.
12	(1.5)
13 PAT	最早也没::提前也没当真.
	zuì zǎo yě méi :: tí qián yě méi dàng zhēn.
	most early either NEG in advance too NEG take seriously.
	*At the very beginning, I didn't take it seriously, either*.
14	那:我觉着现在::看开了.
	nà : wǒ jué zhe xiàn zài :: kàn kāi le.
	that I fell DUR now look open CRS.
	*I think I resign myself to it now*.
15	(0.7)
16 COM	以前也没当真来, (0.4)
	yǐ qián yě méi dàng zhēn lái, (0.4)
	in advance either NEG take seriously come
	*(You said) you didn't take it seriously*.
17 COM	过去几年之前,谁想这个?
	guò qù jǐ nián zhī qián, shuí xiǎng zhè gè?
	past several year before who think this CL
	*Several years before, who would think about this?*
18 DOC	骨质疏松::很厉害啊.
	gǔ zhì shū sōng :: hěn lì hài a.
	osteoporosis very serious PRT.
	*Osteoporosis is very serious*.
19	(0.3)
20 PAT	昂::.
	áng ::.
	*Eh*.

Extract 3 features an elderly woman discussing her back pain related to spinal curvature with the doctor. As she describes the onset of her condition at a transitional relevance place (TRP; [43]), the doctor briefly interrupts to seek confirmation, creating a pause in her narrative (line 5). During this pause, the patient's companion humorously exaggerates the curvature's duration to ‘dozens of years’ (line 7), playfully amplifying the patient's earlier understatement. This humorous correction, highlighted by the companion's additional comment ‘several years before’ (line 17), employs exaggerated terms like ‘hai’ (still) and ‘dei’ (must) to light‐heartedly adjust the patient's description.

This teasing, aligning with the work of Haakana [28, 61, 62] and Jefferson [24], is intended more to amuse than to critique, fostering mutual enjoyment and reinforcing the bond between the patient and her companion. The patient's acceptance of this exaggerated timeframe (line 8) and the ensuing shared loud laughter (lines 8–9) further underscore the tease's benign and nonthreatening nature. In this teasing sequence, the teasable comes from the inadequate answer of the patient in the medical history‐taking sequences, and the tease constitutes a playful correction of the teasable which is met with the patient's joyful acceptance.

Interestingly, the doctor maintaines silence (line 10) after the teasing, allowing the patient space to re‐engage with her narrative. This transition from playful interaction back to a more serious medical discussion is marked by the patient providing a detailed account of her condition after a lengthy gap, which leads to the doctor's medically relevant response about osteoporosis (line 18) [20].

The patient's emphatic self‐blame for her condition (‘ye’ in line 13) and the companion's subsequent reassurance (lines 16–17) illustrate the collaborative nature of teasing in this context, enriching the exploration of the patient's long‐standing condition. The teasing here acts as a catalyst, promoting the patient to provide more detailed information, thus aiding the doctor's understanding of the chronic nature of her condition.

This sequence adeptly navigates between the patient's lived experience, the companion's observations, and the doctor's medical expertise. The companion's playful exaggeration not only injects humour into the dialogue but also prompts a comprehensive discussion about the patient's condition, enhancing the medical narrative and fostering a deeper understanding of her long‐term health issues. Through this interaction, the companion's remarks entertain while reframing the patient's narrative, encouraging a more focused and medically informed discussion.

In this section, we explore the nuanced role of teasing in medical dialogues, emphasizing its effectiveness in exchanging knowledge and facilitating institutional tasks. Through three conversational instances, we demonstrate that teasing is more than just humour; it's a crucial element in the clinical setting, particularly for navigating epistemic territories. Each teasing sequence typically starts with a ‘teasable’—an epistemic claim or medical statement—followed by a ‘tease’ that playfully challenges this claim, and end with a ‘receipt’ where the tease is acknowledged or responded to. These sequences often involve design elements like hypothetical scenarios, mimicry, exaggeration, or mild mockery, often softened by laughter or light‐hearted tones to reduce confrontation. In Extract 1, the doctor employs teasing to address and ease the patient's concerns while imparting crucial information, showcasing how teasing can manage epistemic authority by balancing knowledge sharing with a supportive atmosphere. Extract 2 illustrates a patient using teasing to address conflicting orientations, effectively managing the doctor‐patient relationship by navigating differing perspectives within the consultation. Extract 3 features a companion's tease to prompt the patient to elaborate on her condition, enhancing patient engagement in her health discussion. Overall, teasing in medical settings serves to lighten the atmosphere, and promote knowledge exchange, balancing professional expertise with patient experience.

### 3.2 Managing Deontic Authority

Building on insights from navigating epistemic territories, this section explores teasing's role in the nuanced negotiation of deontic authority within clinician–patient interactions. Teasing not only bridges the gap between expert knowledge and personal experience but also delicately mediates between patient autonomy and professional advice in decision‐making processes. This dynamic is crucial in contexts involving significant health decisions, where teasing emerges as a subtle yet effective tool to probe and negotiate the boundaries of authority and shared responsibility.

In Extract 4, we observe a conversation between a clinician and a patient with lumbago during a medical consultation, further complicated by the presence of a trainee doctor handling electronic tasks. The patient's subsequent suggestion to undertake an X‐ray (line 10) represents a shift from challenging the doctor's knowledge to questioning his decision‐making authority, illustrating how disputes over epistemic authority can escalate into deontic concerns. This progression highlights the intricate dynamics between patient autonomy and professional expertise in medical consultations.

(4) O19612: Strain

**Table:** 

01 DOC	肌肉拉伤啊
	jī ròu lā shāng a.
	muscle strain PRT
	*Muscle strains*.
02	(0.4)
03 → PAT	肌肉拉伤‐光拉伤这儿,
	jī ròu lā shāng ‐ guāng lā shāng zhè ér,
	muscle stain‐ only strain here
	*Muscle strain‐ this place only suffers from*
04 →	骨头没事儿是吧?
	gú tou méi shì ér shì ba?
	bone NEG matter be PRT
	*strained muscle and the bone is fine, right?*
05	(0.4)
06 → DOC	不会恍一下子骨头断下来.
	bú huì huǎng yī xià zi gú tou duàn xià lái.
	NEG can sprain one time bone break down come
	*Your bone won't break when (your waist) is sprained once*.
07	(1.0)
08 → DOC	那这人成了个:
	nà zhè rén chéng le gè :
	that this human become CRS one
	*If so, then this man becomes a*
09	(0.8)
10→ PAT	光:[还得要拍片吧?]
	guāng :[hái dé yào pāi piàn ba?]
	only still have to need shoot film PRT
	*Only: Do I still have to take an X‐ray?*
11→ DOC	[稻草人了.]
	[dào cǎo rén le.]
	Scarecrow CRS.
	scarecrow.
12	(0.5)
13 DOC	不需要拍片子.
	bù xū yào pāi piān zi.
	NEG need shoot film
	*No need to take an X‐ray*.
14 PAT	就是: (0.9)昂.
	jiù shì : (0.9) áng.
	just be (0.9) eh
	*(I'm) just… Eh*.
15	(0.9)
16 → DOC	那恍一下子,那::骨头断了,
	nà huǎng yī xià zi, nà :: gú tou duàn le,
	that sprain one time, that bone break CRS
	*If a man's bone is broken after one sprain*,
17 →	人:不成了稻草人了[吗?
	rén : bù chéng le dào cǎo rén le [ma?
	human NEG become CRS scarecrow CRS PRT
	*then he becomes a scarecrow*.
18 → TRA	[hehhhh.
19	(0.8)
20 PAT	这几天疼的厉‐越来越疼,
	zhè jǐ tiān téng de lì ‐ yuè lái yuè téng,
	this several day pain CSC terri‐ more pain,
	*These days, it hurted terri‐ more and more terribly*,
21	[现在有点儿]怕了,
	[xiàn zài yǒu diǎn ér] pà le,
	[now have a bit] fear PFV,
	*and now I have some fear*.
22 DOC	[昂::.]
	[áng ::.]
	*Eh*.
23 PAT	就一开始想着来,
	jiù yī kāi shǐ xiǎng zhe lái,
	just one beginning think DUR come,
	*In the beginning, I wanted to come (to see a doctor)*,
24	然[后:养着]两天算了吧.
	rán [hòu : yǎng zhe] liǎng tiān suàn le ba.
	then heal DUR two day let it be PRT.
	*but then I thought I just waited for it to heal*.
25 DOC	[涂个:中药.]
	[tú gè : zhōng yào.]
	apply CL herb medicine
	*Apply herb medicine*.
26 PAT	后来不管用.
	hòu lái bù guǎn yòng.
	later NEG effective.
	*But later it didn't work*.
27	(1.1)
28 PAT	早上起床,自己连袜子都穿不来.
	*zǎo shàng qǐ chuáng, zì jǐ lián wà zi dōu chuān bù lái*.
	morning get up self even sock wear NEG come
	*I cannot even wear socks after getting up in the morning*.
29	(3.3)
30 DOC	开XX((name of medicine))软膏抹抹吧.
	kāi XX ruǎn gāo mǒ mǒ ba.
	prescribe XX ointment apply PRT.
	*I will prescribe a XX ointment for you to apply*.
  The sequence begins with the doctor suggesting the patient's symptoms might be due to mere muscle strain (line 1). The patient challenges this assessment, questioning his bone condition and subtly confronting the doctor's initial minor‐problem diagnosis. This counter, framed as a question (lines 3–4), introduces a candidate diagnosis, effectively challenging the doctor's epistemic authority and exerting implicit pressure for a more comprehensive diagnostic explanation [63].
  The doctor's response (line 6) directly addresses the patient's concerns by clarifying that a bone would not break from a sprain, thus reasserting medical authority while engaging with the patient's autonomy. The subsequent silence (line 5) suggests a potential closure of the diagnostic sequence, yet the doctor humorously reengages using a teasing ‘scarecrow’ analogy (lines 8, 11), which downplays the patient's worries while navigating the balance between professional assurance and patient involvement.
  The use of the ‘scarecrow’ analogy lightens the atmosphere and revisits the doctor's medical stance, providing another opportunity to explore the patient's concerns in depth. When the patient suggests an X‐ray (line 10), framed as an inquiry, it poses another challenge to the doctor's authority, indicating a need for further clarification. The doctor's refusal (line 13) initially halts the patient's narrative, highlighting the ongoing negotiation of authority. However, the doctor revisits the ‘scarecrow’ analogy (lines 16–17), transitioning from direct dismissal to a more engaging tease that alleviates the tension and reopens the dialogue, as evidenced by the patient's subsequent detailed sharing of symptoms (lines 23–28).
  The contrast between the doctor's initial refusal and his later teasing, and between the patient's initial halt and later elaboration, underline the efficacy of humour in enhancing patient–doctor communication. This fosters a space where concerns are more openly expressed. During this recounting, the doctor strategically interrupts with a treatment recommendation (line 25) and a prescription (line 30), steering the conversation towards actionable medical steps and underscoring his deontic authority to direct the dialogue.
  Throughout this extract, teasing emerges as a pivotal tool in managing deontic authority, facilitating a nuanced dialogue that respects patient autonomy while ensuring professional guidance. The ‘scarecrow’ analogy not only addresses and mitigates the patient's resistance but also fosters an open dialogue where the patient feels validated and encouraged to delve deeper into their concerns, rather than facing direct contradiction. The diagnosis negotiation and reassurance are navigated through the doctor's adept use of teasing, which, while light‐hearted, significantly contributes to the collaborative decision‐making process.
  Building on the insights from Extract 4, Extract 5 delves deeper into the complexities of decision‐making in patient–doctor interactions, especially those complicated by external factors such as financial constraints. It presents a scenario where an elderly woman must decide between surgical intervention and managing her condition with medication, influenced significantly by her financial situation.
(5) O1960: Rest

**Table:** 

01 DOC	先拿这个药吃吃,是吧？
	xiān ná zhè gè yào chī chī, shì ba？
	first take this CL medicine eat eat be PRT
	*Take the medicine and see how it works first, right?*
02 PAT	嗯.
	en.
	*Mmm*.
03	(0.4)
04 → DOC	歇歇.
	xiē xiē.
	rest rest
	*Have a rest*.
05	(0.8)
06 →COM	heh 什么叫歇啊?不干活了吗?
	*Heh shén me jiào xiē a? bù gàn huó le ma?*
	what call rest PRT? NEG work CRS PRT
	*heh What is rest? No working?*
07	(3.0)
08 → DOC	歇歇,家务活能干干, (0.7)
	xiē xiē, jiā wù huó néng gàn gàn,(0.7)
	rest rest housework can do do
	*Have a rest, and do the housework if you can*.
09	有钱了再‐(.)
	yǒu qián le zài ‐(.)
	have money CRS again
	*When you have enough money*,
10	找我做手术就行了.
	zhǎo wǒ zuò shǒu shù jiù xíng le.
	find I do operation just ok CRS
	*you can come to me to have an operation*
11	把这个腰变得直一些.
	bǎ zhè gè yāo biàn dé zhí yī xiē.
	BA this CL waist become CSC straight some
	*to make your spine straighter*.

In Extract 5, the dialogue begins with the doctor recommending rest, deemed medically appropriate but seemingly impractical for the patient's active lifestyle as a farmer (line 4). The ensuing silence may indicate the patient's hesitation or disagreement, hinting at the advice's impracticality for her daily demands.

The companion starts her turn (line 8) with brief laughter, hearable as a reluctance marker to directly challenge the doctor's advice [64]. She teasingly questions the feasibility of rest by employing exaggerated phrases like ‘no working’, humorously critiquing the advice's lack of practicality [65]. This tease not only highlights the advice's impracticality but also opens a space for negotiation.

This teasing sequence is pivotal, effectively disputing the initial recommendation's applicability, and promoting the doctor to reconsider the advice in light of the patient's real‐world circumstances [66]. The companion's teasing seeks clarification and encourages the doctor to propose a more tailored recommendation that considers the patient's lifestyle and financial limitations, as evidenced in the doctor's repaired response (lines 8–10).

Extract 5 exemplifies the dynamic interplay between medical professionalism and the lived experiences of patients. The companion's teasing, marked by laughter and playful challenge, acts as a crucial mechanism in the decision‐making process, underscoring the necessity for medical recommendations to be clinically sound and adaptable to individual circumstances. This interaction underscores the value of clear, respectful communication in medical consultations, demonstrating how humour can bridge the expertise‐experience gap, leading to more personalized and acceptable medical advice.

In outpatient clinical settings, teasing sequences are integral to decision‐making, typically unfolding in three parts: the ‘teasable’ often stems from a medical diagnosis, decision, or recommendation that may not fully align with the patient's personal or financial considerations; the ‘tease’ humorously challenges this decision, highlighting its potential impracticalities or the patient's reservations; and the ‘receipt’—be it acknowledgement, clarification, modification, or reaffirmation—addresses the concerns raised, ensuring the medical advice remains robust. These exchanges often feature elements of surprise, exaggeration, or irony, with laughter, tone shifts, and specific word choices underscoring the interaction's playful yet serious undertone. Such sequences serve multiple communicative roles: they allow patients or their companions to articulate concerns nonconfrontationally and prompt doctors to reassess or clarify their recommendations. Overall, teasing in decision‐making highlight the institutional contingencies of outpatient interactions, balancing medical authority with patient autonomy, and advocating for collaborative decision‐making. Through playful challenges, these sequences manage potential discord or reluctance, ensuring cooperative interactions. They facilitate the negotiation of institutional goals, ensuring that medical expertise and patient perspectives are both acknowledged and valued.

## 4 Discussion and Conclusion

This study has explored the complex role of teasing within outpatient clinical interactions across various medical phases, including history‐taking, diagnosis, and treatment planning. By analyzing the sequential environment, initiation, design, and interactional tasks of teasing, this research has highlighted its subtle yet significant influence in managing epistemic territories and deontic authority, thereby enriching our understanding of clinician–patient communication dynamics in China.

Teasing, in clinical settings is revealed to operate distinctly from humour and small talk. Unlike humour, which primarily serves to alleviate stress and enhance relational dynamics [8, 9], teasing serves a strategic tool for navigating complex medical dialogues, often emerging from discord between patient expectations and the clinician's narrative [19]. This divergence from everyday teasing scenarios, where teasing may involve exaggerating or persistently focusing on a subject, underscores its tailored use in healthcare.

Comparatively, small talk in medical interactions generally involves exchanges that are topically distinct from the main institutional agenda but play a crucial role in easing into more substantive discussions, establishing rapport, and making the clinical environment less intimidating [11]. Small talk often precedes or follows clinical discussions, serving as a buffer and facilitating smoother transitions between different phases of the medical interaction [10, 12]. However, unlike small talk, teasings are often directly integrated with the medical content, uniquely positioned to challenge or reaffirm medical perspectives in a light‐hearted yet impactful manner [54].

The nuanced use of teasing compared to humour and small talk reflects a sophisticated understanding of conversational dynamics within clinical settings. Teasing can bridge the gap between professional medical advice and patient receptivity, subtly influencing patient behaviour and expectations through its dual role of challenging and affirming. This is unlike small talk, which primarily eases social interactions without directly engaging with medical content, and humour, which may not always carry the epistemic or deontic weight that teasing does in clinical discourse.

The concept of ‘teasable’ moments within medical consultations highlights how specific statements or behaviours become targets for teasing based on their dynamics and interactional nature. These moments are pivotal for clinicians to navigate complex emotional and informational landscapes effectively. For instance, clinicians in Extracts 1 and 4 use teasing to alleviate tension, simplify medical information, or address misconceptions, enhancing patient engagement without compromising professional integrity. Conversely, patients and companions in Extract 2 and 5 may use teasing to subtly challenge medical advice or express concerns, providing clinicians with insights into the patient's emotional and social context. Teasing between patients and companions typically reflects their close relationship and shared experiences, serving as mutual support and an emotional coping mechanism amid medical challenges. These instances highlight the subtle ways in which certain aspects of clinical dialogue are more prone to being teased, reflecting the inherent flexibility and adaptability of conversational practices in outpatient settings.

Moreover, the sensitivity required in the use of teasing reflects a crucial aspect of medical professionalism. Effective use of teasing requires understanding its potential impacts—when it might comfort or distress, which is essential for maintaining the therapeutic alliance, underscoring the need for training programs that enhance clinicians' skills in employing humour effectively [19, 50, 67, 68]. This is particularly important in light of studies such as by Haakana [28], who demonstrates the role of laughter and humour in managing patients' experiences of illness.

Overall, this study underscores the intricate role of teasing in managing both epistemic and deontic authority within the clinician‐patient relationship. By weaving together humour with seriousness, teasing not only enhances communication efficacy but also enriches the patient‐healthcare provider dialogue, ensuring that it remains empathetic, and patient‐centred. Future research should expand on this foundational work by exploring a wider array of clinical settings among different participants and interactions to fully capture the spectrum of teasables and its impacts on clinical communication dynamics, and by employing multimodal analyses that include visual data. Such expansions could enrich our understanding of how nonverbal cues such as facial expressions and body language contribute to the dynamics of teasing in clinical interactions.

In conclusion, teasing in outpatient clinical interactions is a multifaceted communicative practice that balances professionalism with empathy. It plays a pivotal role in both managing medical authority and enhancing patient engagement, thus contributing to more effective and satisfying healthcare experiences. Further exploration into the strategic use of teasing within these interactions can lead to better communication strategies that support both the relational and instrumental goals of clinical practice.

## Author Contributions

**Ying Gao:** conceptualization, methodology, data curation, investigation, formal analysis, writing—original draft, project administration. **Shuai Zhang:** investigation, formal analysis, writing—review and editing. **Wen Ma:** conceptualization, funding acquisition; formal analysis, investigation, writing—review and editing.

## Conflicts of Interest

The authors declare no conflicts of interest.

## Supporting information

Supporting information.

## Data Availability

The data that support the findings of this study are available on request from the corresponding author. The data are not publicly available due to privacy or ethical restrictions.

## References

[1] J. F. Ha and N. Longnecker , “Doctor–Patient Communication: A Review,” Ochsner Journal 10 (2010): 38–43. 21603354 PMC3096184

[2] S. Mace , S. Collins , and S. Speer , “Talking About Breast Symmetry in the Breast Cancer Clinic: What Can We Learn From an Examination of Clinical Interaction,” Health Expectations 24 (2021): 209–221. 33517586 10.1111/hex.13144 PMC8077149

[3] E. Forsgren and I. Björkman , “Interactional Practices in Person‐Centred Care: Conversation Analysis of Nurse‐Patient Disagreement During Self‐Management Support,” Health Expectations 24 (2021): 940–950. 33774894 10.1111/hex.13236 PMC8235886

[4] K. Grainger , “‘That's a Lovely Bath Dear’: Reality Construction in the Discourse of Elderly Care,” Journal of Aging Studies 7 (1993): 247–262.

[5] W. A. Beach and E. Prickett , “Laughter, Humor, and Cancer: Delicate Moments and Poignant Interactional Circumstances,” Health Communication 32, no. 7 (2017): 791–802. 27420294 10.1080/10410236.2016.1172291

[6] J. Kors , A. de la Croix , L. Martin , et al., “Autonomy‐Supportive Decision‐Making in Maternity Care During Prenatal Consultations: A Qualitative Interaction Analysis,” BMJ Open 12 (2022): e063463. 10.1136/bmjopen-2022-063463 PMC9670942 36385034

[7] J. Mallett and R. A'hern , “Comparative Distribution and Use of Humour Within Nurse‐Patient Communication,” International Journal of Nursing Studies 33 (1996): 530–550. 8886903 10.1016/0020-7489(96)00008-9

[8] A. C. Schöpf , G. S. Martin , and M. A. Keating , “Humor as a Communication Strategy in Provider‐Patient Communication in a Chronic Care Setting,” Qualitative Health Research 27, no. 3 (2017): 374–390. 26717942 10.1177/1049732315620773

[9] U. Warner , “The Serious Import of Humour in Health Visiting,” Journal of Advanced Nursing 9, no. 1 (1984): 83–87. 6561220 10.1111/j.1365-2648.1984.tb00347.x

[10] P. L. Hudak and D. W. Maynard , “An Interactional Approach to Conceptualising Small Talk in Medical Interactions,” Sociology of Health & Illness 33 (2011): 634–653. 21545445 10.1111/j.1467-9566.2011.01343.x PMC3609551

[11] B. Benwell and M. McCreaddie , “Keeping “Small Talk” Small in Health‐Care Encounters: Negotiating the Boundaries Between On‐ and Off‐Task Talk,” Research on Language and Social Interaction 49, no. 3 (2016): 258–271.

[12] D. W. Maynard and P. L. Hudak , “Small Talk, High Stakes: Interactional Disattentiveness in the Context of Prosocial Doctor‐Patient Interaction,” Language in Society 37, no. 5 (2008): 661–688.

[13] A. Kwame and P. M. Petrucka , “A Literature‐Based Study of Patient‐Centered Care and Communication in Nurse‐Patient Interactions: Barriers, Facilitators, and the Way Forward,” BMC Nursing 20 (2021): 158. 34479560 10.1186/s12912-021-00684-2 PMC8414690

[14] J. Ruan , Guanxi, Social Capital and School Choice in China: The Rise of Ritual Capital (London: Springer International Publishing, 2017).

[15] N. C. Wang , “Claiming or Abdicating Medical Authority: Treatment Recommendation Actions, Doctor‐Patient Relationship, and Antibiotic Overprescription in Chinese Paediatrics,” Sociology of Health & Illness 46 (2024): 722–743, 10.1111/1467-9566.13733. 38063484

[16] C. Zhao and W. Ma , “Patient Resistance Towards Clinicians' Diagnostic Test‐Taking Advice and Its Management in Chinese Outpatient Clinic Interaction,” Social Science & Medicine (1982) 258 (2020): 113041. 32480183 10.1016/j.socscimed.2020.113041

[17] M. Haugh , “Jocular Mockery, (Dis)affiliation and Face,” Journal of Pragmatics 42, no. 8 (2010): 2106–2119.

[18] S. D. Looney , “Classroom Teasing: Institutional Contingencies and Embodied Action,” Discourse Studies 23, no. 4 (2021): 519–538.

[19] P. Drew , “Po‐Faced Receipts of Teases,” Linguistics 25, no. 1 (1987): 219–253.

[20] E. A. Schegloff , Sequence Organization in Interaction (Cambridge: Cambridge University Press, 2007).

[21] C. Li , “Teasing as a Practice of Managing Delicate Issues in Institutional Talk: A Case Study of Request in Mandarin Chinese,” East Asian Pragmatics 5, no. 3 (2020): 323–344.

[22] N. Lilja and S. W. Eskildsen , “The Embodied Work of Repairing‐for‐Teasing in Everyday L2 Talk,” Social Interaction. Video‐Based Studies of Human Sociality 5, no. 1 (2022), 10.7146/si.v5i2.130872.

[23] P. Glenn , Laughter in Interaction (Cambridge: Cambridge University Press, 2003).

[24] G. Jefferson , “On Stepwise Transition From Talk About a Trouble to Inappropriately Next‐Positioned Matters,” in Structures of Social Action: Studies in Conversation Analysis, eds. M. Atkinson and J. Heritage (Cambridge: Cambridge University Press, 1984). 191–222.

[25] S. Schnurr , “Constructing Leader Identities Through Teasing at Work,” Journal of Pragmatics 41, no. 6 (2009): 1125–1138.

[26] S. Schnurr and A. Chan , “When Laughter Is Not Enough. Responding to Teasing and Self‐Denigrating Humour at Work,” Journal of Pragmatics 43, no. 1 (2011): 20–35.

[27] T. Auburn and C. Pollock , “Laughter and Competence: Children With Severe Autism Using Laughter to Joke and Tease,” in Studies of Laughter in Interaction, eds. P. Glenn and E. Holt (London: Bloomsbury Academic, 2013), 135–160.

[28] M. Haakana , “Laughter as a Patient's Resource: Dealing With Delicate Aspects of Medical Interaction,” Text 21, no. 1/2 (2001): 187–219.

[29] E. Holt , “The Last Laugh: Shared Laughter and Topic Termination,” Journal of Pragmatics 42, no. 6 (2010): 1513–1525.

[30] J. Heritage , “The Epistemic Engine: Sequence Organization and Territories of Knowledge,” Research on Language and Social Interaction 45 (2012): 25–50.

[31] J. Heritage , “Epistemics in Action: Action Formation and Territories of Knowledge,” Research on Language and Social Interaction 45 (2012): 1–25.

[32] J. Heritage , “Epistemics in Conversation,” in Handbook of Conversation Analysis, eds. J. Sidnell and T. Stivers (Chichester: Wiley‐Blackwell, 2013). 370–394.

[33] M. Stevanovic and A. Peräkylä , “Deontic Authority in Interaction: The Right to Announce, Propose, and Decide,” Research on Language & Social Interaction 45, no. 3 (2012): 297–321.

[34] J. Heritage and G. Raymond , “The Terms of Agreement: Indexing Epistemic Authority and Subordination in Talk‐in‐Interaction,” Social Psychology Quarterly 68, no. 1 (2005): 15–38.

[35] A. M. D. Landmark , P. Gulbrandsen , and J. Svennevig , “Whose Decision? Negotiating Epistemic and Deontic Rights in Medical Treatment Decisions,” Journal of Pragmatics 78 (2015): 54–69.

[36] A. Lindström and A. Weatherall , “Orientations to Epistemics and Deontics in Treatment Discussions,” Journal of Pragmatics 78 (2015): 39–53.

[37] M. Jakovljevic , H. Chang , J. Pan , et al., “Successes and Challenges of China's Health Care Reform: A Four‐Decade Perspective Spanning 1985–2023,” Cost Effectiveness and Resource Allocation 21, no. 1 (2023): 59. 37649062 10.1186/s12962-023-00461-9 PMC10469830

[38] G. M. Fix , C. VanDeusen Lukas , R. E. Bolton , et al., “Patient‐Centred Care Is a Way of Doing Things: How Healthcare Employees Conceptualize Patient‐Centred Care,” Health Expectations 21, no. 1 (2018): 300–307. 28841264 10.1111/hex.12615 PMC5750758

[39] G. Jefferson , “An Exercise in the Transcription and Analysis of Laughter,” in Handbook of Discourse Analysis, ed. T. A. van Dijk (New York: Academic Press, 1985). 25–34.

[40] G. Jefferson , “Glossary of Transcript Symbols With an Introduction,” in Conversation Analysis: Studies from the First Generation, ed. G. Lerner (New York: John Benjamins, 2004). 13–31.

[41] J. Sidnell and T. Stivers , eds., Handbook of Conversation Analysis (Chichester: Wiley‐Blackwell, 2012).

[42] I. Hutchby and R. Wooffitt , Conversation Analysis: Principles, Practices and Applications (Cambridge: Polity Press, 1998).

[43] H. Sacks , E. A. Schegloff , and G. Jefferson , “A Simplest Systematics for the Organization of Turn‐Taking for Conversation,” Language 50, no. 4 (1974): 696–735.

[44] E. A. Schegloff , “Between Micro and Macro: Contexts and Other Connections,” in The Micro‐Macro Link, eds. J. Alexander , B. Giesen , R. Munch and N. Smelser (University of California Press, 1987), 207–234.

[45] E. A. Schegloff , “Reflections on Talk and Social Structure,” in Talk and Social Structure: Studies in Ethnomethodology and Conversation Analysis, eds. D. Boden and D. H. Zimmerman (Cambridge: Polity Press, 1991). 44–70.

[46] E. A. Schegloff , “In Another Context,” in Rethinking Context: Language as an Interactive Phenomenon, eds. A. Duranti and C. Goodwin (Cambridge: Cambridge University Press, 1992). 193–227.

[47] E. A. Schegloff , “Whose Text? Whose Context?” Discourse & Society 8, no. 2 (1997): 165–187.

[48] E. A. Schegloff , “Overlapping Talk and the Organization of Turn‐Taking for Conversation,” Language in Society 29, no. 1 (2000): 1–63.

[49] S. Bloch and C. Antaki , “How Professionals Deal With Clients' Explicit Objections to Their Advice,” Discourse Studies 24, no. 4 (2022): 385–403.

[50] T. Stivers , J. Heritage , R. K. Barnes , R. McCabe , L. Thompson , and M. Toerien , “Treatment Recommendations as Actions,” Health Communication 33, no. 11 (2018): 1335–1344. 28816510 10.1080/10410236.2017.1350913

[51] R. Wilkinson , “Commentary—Developing the Comparative Perspective in Atypical Interaction Research,” Clinical Linguistics & Phonetics 34, no. 10–11 (2020): 1045–1054. 32657161 10.1080/02699206.2020.1786604

[52] R. K. Barnes , “Conversation Analysis of Communication in Medical Care: Description and Beyond,” Research on Language and Social Interaction 52, no. 3 (2019): 300–315.

[53] V. Land , R. Parry , and J. Seymour , “Communication Practices That Encourage and Constrain Shared Decision Making in Health‐Care Encounters: Systematic Review of Conversation Analytic Research,” Health Expectations 20, no. 6 (2017): 1228–1247. 28520201 10.1111/hex.12557 PMC5690232

[54] J. Heritage and D. W. Maynard , Communication in Medical Care: Interaction between Primary Care Physicians and Patients (Cambridge: Cambridge University Press, 2006).

[55] M. Pino , “Hurting and Blaming: Two Components in the Action Formation of Complaints About Absent Parties,” Research on Language & Social Interaction 55, no. 3 (2022): 260–278.

[56] E. A. Schegloff , “Ten Operations in Self‐Initiated, Same‐Turn Repair.” in Conversational Repair and Human Understanding, eds. M. Hayashi , G. Raymond and J. Sidnell (Cambridge: Cambridge University Press, 2013). 69–111.

[57] A. Pomerantz , “Compliment Responses: Notes on the Co‐operation of Multiple Constraints,” in Studies in the Organization of Conversational Interaction, ed. J. Schenkein (New York: Academic Press, 1978). 79–112.

[58] J. D. Robinson , “Accountability in Social Interaction,” in Accountability in Social Interaction, ed. J. D. Robinson (Oxford: Oxford University Press, 2016). 1–44.

[59] J. P. Emerson , “Negotiating the Serious Import of Humor,” Sociometry 32 (1969): 169–181.

[60] M. Fatigante and F. Orletti “Laughter and Smiling in a Three‐Party Medical Encounter: Negotiating Participants' Alignment in Delicate Moments,” in Studies of Laughter in Interaction, eds. P. Glenn and E. Holt (London: Bloomsbury Academic, 2013). 161–183.

[61] M. Haakana , “Laughter in Medical Interaction: From Quantification to Analysis, and Back,” Journal of Sociolinguistics 6, no. 2 (2002): 207–235.

[62] M. Haakana , “Laughter and Smiling: Notes on Co‐Occurrences,” Journal of Pragmatics 42 (2010): 1499–1512.

[63] T. Stivers , “Presenting the Problem in Pediatric Encounters: “Symptoms Only” Versus “Candidate Diagnosis” Presentations,” Health Communication 14, no. 3 (2002): 299–338. 12186491 10.1207/S15327027HC1403_2

[64] A. Pomerantz , “Agreeing and Disagreeing With Assessments: Some Features of Prefered/Dispreferred Turn Shapes,” in Structures of Social Action: Studies in Conversation Analysis, eds. J. M. Atkinson and J. Heritage (Cambridge: Cambridge University Press, 1984). 57–101.

[65] A. Pomerantz , “Extreme Case Formulations: A Way of Legitimizing Claims,” Human Studies 9 (1986): 219–229.

[66] G. Jefferson and J. R. E. Lee , “The Rejection of Advice: Managing the Problematic Convergence of a ‘Troubles‐Telling’ and a ‘Service Encounter’,” Journal of Pragmatics 5, no. 5 (1981): 399–422.

[67] T. Stivers and R. K. Barnes , “Treatment Recommendation Actions, Contingencies, and Responses: An Introduction,” Health Communication 33, no. 11 (2018): 1331–1334. 28825505 10.1080/10410236.2017.1350914

[68] M. Toerien , “When Do Patients Exercise Their Right to Refuse Treatment? A Conversation Analytic Study of Decision‐Making Trajectories in UK Neurology Outpatient Consultations,” Social Science & Medicine 290 (2021): 114278. 34373128 10.1016/j.socscimed.2021.114278

