# Supplementary material for: Teasing in Outpatient Clinical Interaction in China: Managing Epistemic and Deontic Authorities
Source: Health Expect. 2024 Jun 21;27(3):e14114. doi: 10.1111/hex.14114 (PMC11192843; doi:10.1111/hex.14114)
Supplement: Supplementary file 1 — Supporting information. [file HEX-27-e14114-s001.docx]

# Data

1. O1957: Crying loudly

| 01 → PAT | 你把这个还得-(.) |
| --- | --- |
|  | nǐ bǎ zhè gè hái déi -(.) |
|  | you BA this CL still have to |
|  | *You still want (to keep) it -* |
| 02 → | 如果不拆的话,得带多长时间? |
|  | rú guǒ bù chāi de huà , dé dài duō zhǎng shí jiān ? |
|  | if NEG remove CSC talk have to wear how long time |
|  | *If it is not removed, how long will (I) wear it?* |
| 03 | (0.7) |
| 04 → DOC | 是不是当时你光-在那里嗷嗷的在 |
|  | shì bú shì dāng shí nǐ guāng - zài nà lǐ áo áo de zài |
|  | be NEG be then you only at there ao ao CSC at |
|  | *Were you just there going ‘ao ao’, wailing away,* |
| 05 → | 那里哭,哄你说的三-三个月啊. |
|  | nà lǐ kū,hǒng nǐ shuō de sān - sān gè yuè a. |
|  | there cry coax you say CSC three three CL month PRT. |
|  | *and they coaxed you by saying ‘three months’, right?* |
| 06 | (0.4) |
| 07 → PAT | 哭倒没哭.疼[啊. |
|  | kū dào méi kū . téng [ a . |
|  | cry actually NEG cry. pain PRT. |
|  | *I didn’t cry, through. It [pained.* |
| 08 DOC | [你不是疼吗? |
|  | [ nǐ bú shì téng ma ? |
|  | [you NEG be pain PRT |
|  | [*Didn’t you feel painful?* |
| 09 | (0.3) |
| 10 PAT | 疼我也不哭. |
|  | téng wǒ yě bù kū . |
|  | pain I either NEG cry |
|  | *Even it pained, I didn’t cry.* |
| 11 | (0.3) |
| 12 DOC | 不哭,你-你你你:如果要是:(0.2) |
|  | bù kū , nǐ - nǐ nǐ nǐ : rú guǒ yào shì :（0.2） |
|  | NEG cry you you you you if suppose |
|  | *You didn’t cry. If -* |
| 13 | 如果要是说就是:不是这个的话, |
|  | rú guǒ yào shì shuō jiù shì : bú shì zhè gè de huà , |
|  | If suppose say just be NEG be this CL ASSC talk, |
|  | *If it was not the case,* |
| 14 | 三个月那是不对的哈.（.） |
|  | sān gè yuè nà shì bú duì de hā .（.） |
|  | three CL month that be NEG right ASSC PRT |
|  | *then three-month is not correct.* |
| 15 | 绝对不对的.半年都不对. |
|  | jué duì bú duì de . bàn nián dōu bú duì . |
|  | definitely NEG right ASSC half year all NEG right. |
|  | *Definitely not correct. Even half a year is not correct.* |
| 16 | (0.8) |
| 17 PAT | 这十个[月了, |
|  | zhè shí gè [ yuè le , |
|  | this ten CL month PFV. |
|  | *It has been ten months.* |
| 18 DOC | [你-给你造成了 |
|  | [ nǐ - gěi nǐ zào chéng le |
|  | [you- give you cause PFV |
| 19 | 一个很大的误导啊,这是. |
|  | yí gè hěn dà de wù dǎo a , zhè shì . |
|  | one CL very big ASSC misleading PRT, this be. |
|  | *This misled you a lot.* |

1. O1957-02: Knead

| 01 → DOC | 不疼吧? |
| --- | --- |
|  | bù téng ba ? |
|  | NEG pain PRT? |
|  | *It doesn’t pain, right?* |
| 02 | (0.7) |
| 03 PAT | 肯定有点疼,但是不是很疼. |
|  | kěn dìng yǒu diǎn téng , dàn shì bú shì hěn téng . |
|  | definitely have a bit pain, but NEG be very painful. |
|  | *It must hurt in some way, but it is not very painful.* |
| 04 | (1.0) |
| 05 → PAT | 叫-你叫好腿你捏, |
|  | jiào-nǐ jiào hǎo tuǐ nǐ niē, |
|  | ask you ask good leg you knead, |
|  | *If you knead an intact leg,* |
| 06→ | 你也疼啊, £对吧? £ |
|  | nǐ yě téng a ,£duì ba?£ |
|  | you too hurt PRT, right PRT? |
|  | *it will also hurt, £right?£* |
| 07 → DOC | 不一个疼法. |
|  | bù yī gè téng fǎ . |
|  | NEG one CL hurt way. |
|  | *It hurts in a different way.* |
| 08 | (1.8) |

1. O1915: Dozens of years

| 01 DOC | 这种wa-歪歪着, (0.3)多长时间啦? |
| --- | --- |
|  | zhè zhǒng wa- wāi wāi zhe , (0.3) duō zhǎng shí jiān la ? |
|  | This kind cur- curved DUR (0.3) how long time PFV |
|  | *How long is the curvature (of the spine)?* |
| 02 → PAT | 歪歪着,好几年啦.[在我 |
|  | wāi wāi zhe , hǎo jǐ nián la .[ zài wǒ |
|  | curved DUR great several year PFV at I |
|  | *The curvature has been several years. When I (was)…* |
| 03 DOC | [好几年啦? |
|  | [hǎo jǐ nián la ? |
|  | [great several year PFV |
|  | *Several years?* |
| 04 PAT | 昂. |
|  | áng . |
|  | *Eh.* |
| 05 | (0.6) |
| 06 → COM | 还好几年了, |
|  | hái hǎo jǐ nián le , |
|  | still great several year PFV, |
|  | *It cannot be several years.* |
| 07 → | 这得好几十年. |
|  | zhè déi hǎo jǐ shí nián . |
|  | this must great several ten year. |
|  | *It has to be dozens of years.* |
| 08 → PAT | 昂[:.好几十::]年£是[不假哩.] £ |
|  | áng [:. hǎo jǐ shí ::] nián £ shì [ bù jiǎ lī .] £ |
|  | eh great several ten year be N fake PRT |
|  | *Eh. Dozens of years.* £*It’s not fake.* £ |
| 09 → COM | [↑Hah hehhh.] [↑Hah heheh]h. |
| 10 | (1.5) |
| 11 PAT | 非锅锅着,这个腰恁. |
|  | fēi guō guō zhe , zhè gè yāo nèn . |
|  | must arch DUR this CL waist PRT. |
|  | *The back must be stooped itself.* |
| 12 | (1.5) |
| 13 PAT | 最早也没::提前也没当真. |
|  | zuì zǎo yě méi :: tí qián yě méi dàng zhēn . |
|  | most early either NEG in advance too NEG take seriously. |
|  | *At the very beginning, I didn’t take it seriously, either.* |
| 14 | 那:我觉着现在::看开了. |
|  | nà : wǒ jué zhe xiàn zài :: kàn kāi le . |
|  | that I fell DUR now look open CRS. |
|  | *I think I resign myself to it now.* |
| 15 | (0.7) |
| 16 COM | 以前也没当真来, (0.4) |
|  | yǐ qián yě méi dàng zhēn lái , (0.4) |
|  | in advance either NEG take seriously come |
|  | *(You said) you didn’t take it seriously.* |
| 17 COM | 过去几年之前,谁想这个? |
|  | guò qù jǐ nián zhī qián , shuí xiǎng zhè gè ? |
|  | past several year before who think this CL |
|  | *Several years before, who would think about this?* |
| 18 DOC | 骨质疏松::很厉害啊. |
|  | gǔ zhì shū sōng :: hěn lì hài a . |
|  | osteoporosis very serious PRT. |
|  | *Osteoporosis is very serious.* |
| 19 | (0.3) |
| 20 PAT | 昂::. |
|  | áng ::. |
|  | *Eh.* |

1. O19612: Strain

| 01 DOC | 肌肉拉伤啊 |
| --- | --- |
|  | jī ròu lā shāng a. |
|  | muscle strain PRT |
|  | *Muscle strains.* |
| 02 | (0.4) |
| 03 → PAT | 肌肉拉伤-光拉伤这儿, |
|  | jī ròu lā shāng - guāng lā shāng zhè ér , |
|  | muscle stain- only strain here |
|  | *Muscle strain- this place only suffers from* |
| 04 → | 骨头没事儿是吧? |
|  | gú tou méi shì ér shì ba ? |
|  | bone NEG matter be PRT |
|  | *strained muscle and the bone is fine, right?* |
| 05 | (0.4) |
| 06 → DOC | 不会恍一下子骨头断下来. |
|  | bú huì huǎng yī xià zi gú tou duàn xià lái . |
|  | NEG can sprain one time bone break down come |
|  | *Your bone won’t break when (your waist) is sprained once.* |
| 07 | (1.0) |
| 08 → DOC | 那这人成了个: |
|  | nà zhè rén chéng le gè : |
|  | that this human become CRS one |
|  | *If so, then this man becomes a* |
| 09 | (0.8) |
| 10→ PAT | 光:[还得要拍片吧?] |
|  | guāng :[ hái dé yào pāi piàn ba ?] |
|  | only still have to need shoot film PRT |
|  | *Only: Do I still have to take an X-ray?* |
| 11→ DOC | [稻草人了.] |
|  | [ dào cǎo rén le .] |
|  | Scarecrow CRS. |
|  | scarecrow. |
| 12 | (0.5) |
| 13 DOC | 不需要拍片子. |
|  | bù xū yào pāi piān zi . |
|  | NEG need shoot film |
|  | *No need to take an X-ray.* |
| 14 PAT | 就是: (0.9)昂. |
|  | jiù shì : (0.9) áng. |
|  | just be (0.9) eh |
|  | *(I’m) just… Eh.* |
| 15 | (0.9) |
| 16 → DOC | 那恍一下子,那::骨头断了, |
|  | nà huǎng yī xià zi , nà :: gú tou duàn le , |
|  | that sprain one time, that bone break CRS |
|  | *If a man’s bone is broken after one sprain,* |
| 17 → | 人:不成了稻草人了[吗? |
|  | rén : bù chéng le dào cǎo rén le [ ma ? |
|  | human NEG become CRS scarecrow CRS PRT |
|  | *then he becomes a scarecrow.* |
| 18 → TRA | [hehhhh. |
| 19 | (0.8) |
| 20 PAT | 这几天疼的厉-越来越疼, |
|  | zhè jǐ tiān téng de lì - yuè lái yuè téng , |
|  | this several day pain CSC terri- more pain, |
|  | *These days, it hurted terri- more and more terribly,* |
| 21 | [现在有点儿]怕了, |
|  | [ xiàn zài yǒu diǎn ér ] pà le , |
|  | [now have a bit] fear PFV, |
|  | *and now I have some fear.* |
| 22 DOC | [昂::.] |
|  | [ áng ::.] |
|  | *Eh.* |
| 23 PAT | 就一开始想着来, |
|  | jiù yī kāi shǐ xiǎng zhe lái , |
|  | just one beginning think DUR come, |
|  | *In the beginning, I wanted to come (to see a doctor),* |
| 24 | 然[后:养着]两天算了吧. |
|  | rán [ hòu : yǎng zhe ] liǎng tiān suàn le ba . |
|  | then heal DUR two day let it be PRT. |
|  | *but then I thought I just waited for it to heal.* |
| 25 DOC | [涂个:中药.] |
| [ tú gè : zhōng yào .] |  |
|  | apply CL herb medicine |
|  | *Apply herb medicine.* |
| 26 PAT | 后来不管用. |
|  | hòu lái bù guǎn yòng . |
|  | later NEG effective. |
|  | *But later it didn’t work.* |
| 27 | (1.1) |
| 28 PAT | 早上起床,自己连袜子都穿不来. |
| *.* | *zǎo shàng qǐ chuáng, zì jǐ lián wà zi dōu chuān bù lái* |
|  | morning get up self even sock wear NEG come |
|  | *I cannot even wear socks after getting up in the morning.* |
| 29 | (3.3) |
| 30 DOC | 开XX((name of medicine))软膏抹抹吧. |
|  | kāi XX ruǎn gāo mǒ mǒ ba . |
|  | prescribe XX ointment apply PRT. |
|  | *I will prescribe a XX ointment for you to apply.* |

1. O1960: Rest

| 01 DOC | 先拿这个药吃吃,是吧？ |
| --- | --- |
|  | xiān ná zhè gè yào chī chī , shì ba ？ |
|  | first take this CL medicine eat eat be PRT |
|  | *Take the medicine and see how it works first, right?* |
| 02 PAT | 嗯. |
|  | en. |
|  | *Mmm.* |
| 03 | (0.4) |
| 04 → DOC | 歇歇. |
|  | xiē xiē . |
|  | rest rest |
|  | *Have a rest.* |
| 05 | (0.8) |
| 06 →COM | heh 什么叫歇啊?不干活了吗? |
|  | *Heh shén me jiào xiē a ? bù gàn huó le ma ?* |
|  | what call rest PRT? NEG work CRS PRT |
|  | *heh What is rest? No working?* |
| 07 | (3.0) |
| 08 → DOC | 歇歇,家务活能干干, (0.7) |
|  | xiē xiē , jiā wù huó néng gàn gàn ,(0.7) |
|  | rest rest housework can do do |
|  | *Have a rest, and do the housework if you can.* |
| 09 | 有钱了再-(.) |
|  | yǒu qián le zài -(.) |
|  | have money CRS again |
|  | *When you have enough money,* |
| 10 | 找我做手术就行了. |
|  | zhǎo wǒ zuò shǒu shù jiù xíng le . |
|  | find I do operation just ok CRS |
|  | *you can come to me to have an operation* |
| 11 | 把这个腰变得直一些. |
|  | bǎ zhè gè yāo biàn dé zhí yī xiē . |
|  | BA this CL waist become CSC straight some |
|  | *to make your spine straighter.* |

# Appendix

## Transcription symbols

The transcription symbols used in this article are modified from Jefferson (1985, 2004):

[ a point where overlapping speech occurs

] a point where overlapping speech ends

(0.7) a timed pause.

(.) a micro-pause (1 tenth of a second or less)

:: prolongation or stretching of the sound just preceding them

, a slightly rising intonation, or ‘continuing’ intonation

. a falling intonation

? a rising intonation (not necessarily a question)

underline a raise in volume or emphasis

hhh hearable aspiration (may represent breathing, laughter, etc.)

.hhh in-breath

- a cut-off or self-interruption.

(( )) description of the situation or notes

£ £ talk between pound symbols is spoken with laughter

## Glossing abbreviations

ASSC associative

BA ba (a marker for object promotion)

CRS currently relevant state (*le*)

CSC complex stative construction

CL classifier

DUR durative aspect(-*zhe*)

NEG negation marker

PRT particle

PFV perfective aspect(-*le*)
